# Preventive effect of *Dendrobium candidum* Wall. ex Lindl. on activated carbon-induced constipation in mice

**DOI:** 10.3892/etm.2014.2119

**Published:** 2014-12-08

**Authors:** RUI WANG, PENG SUN, YALIN ZHOU, XIN ZHAO

**Affiliations:** Department of Biological and Chemical Engineering, Chongqing University of Education, Chongqing 400067, P.R. China

**Keywords:** *Dendrobium candidum*, activated carbon, constipation, gastrointestinal transit, mice

## Abstract

The aim of this study was to investigate the effects of *Dendrobium candidum* Wall. ex Lindl. (*D. candidum*) on activated carbon-induced constipation in ICR mice. ICR mice were orally administered *D. candidum* for 9 days. Body weight, defecation status, gastrointestinal (GI) transit and defecation times, in addition to the levels of motilin (MTL), gastrin (Gas), endothelin (ET), somatostatin (SS), acetylcholinesterase (AChE), substance P (SP) and vasoactive intestinal peptide (VIP) in serum were used to evaluate the preventive effects of *D. candidum* on constipation. The laxative drug bisacodyl acted as a positive control. The time to the first defecation of a black stool for the normal, control, bisacodyl-treated (100 mg/kg), 200 and 400 mg/kg *D. candidum*-treated mice was 84, 202, 126, 161 and 142 min, respectively. Following the consumption of 200 and 400 mg/kg *D. candidum* or bisacodyl (100 mg/kg), the GI transit was reduced to 57.7, 74.6 and 90.2%, respectively, of the transit in normal mice. The serum levels of MTL, Gas, ET, AChE, SP and VIP were significantly increased and the serum levels of SS were reduced in the mice treated with *D. candidum* compared with those in the untreated control mice (P<0.05). These results demonstrate that *D. candidum* has preventive effects on constipation in mice, and a greater functional activity was observed when a higher concentration was administered.

## Introduction

*Dendrobium* is a huge genus of orchids, and *Dendrobium candidum* Wall. ex Lindl. (*D. candidum*) is a synonym of *Dendrobium moniliforme* (L.) Sw. (1,2). It is a traditional Chinese medicinal herb that is used raw or processed for health care products in China (3). *D. candidum* contains water-soluble polysaccharides, phenanthrenes and many amino acids. It also has high contents of chrysotoxen and erianin, which may have medical effects (4).

Constipation is defined medically as the passing of fewer than three stools per week and is considered severe when there is less than one stool per week. It may occur when the colon absorbs too much water (5). In the current study, activated carbon was orally administered to mice to generate a model of constipation. Activated carbon attaches to the mucosal surfaces of the gastrointestinal (GI) tract and reduces drainage. This causes a reduction of GI fluids and slows down GI movement, resulting in weakness of the spleen and stomach, and ultimately to constipation.

The model of constipation induced by activated carbon has been used to demonstrate the effects of drugs in the treatment of constipation (6,7). A megadose of activated carbon has been shown to result in digestive tract obstruction (8). In the present study, the functional effects of *D. candidum* in the alimentary tract were investigated in a mouse model of activated carbon-induced constipation. GI transit, time to the first defecation of a black stool, and serum levels of motilin (MTL), gastrin (Gas), endothelin (ET), somatostatin (SS), acetylcholinesterase (AChE), substance P (SP) and vasoactive intestinal peptide (VIP) were examined. Bisacodyl, a laxative drug that stimulates intestinal peristalsis and acts directly on the colon to produce a bowel movement, was used as a positive control. Bisacodyl is typically prescribed for the relief of constipation and for the management of neurogenic bowel dysfunction, as well as for bowel preparation prior to medical examinations (9–11).

The current study was designed to investigate the anti-constipation effects of *D. candidum* on activated carbon-induced constipation and also to elucidate the association of its protective effects with the chemical compounds it contains.

## Materials and methods

### Preparation of D. candidum

A sample of *D. candidum* was purchased from Shanghai Pharmacy Co., Ltd. (Shanghai, China). The *D. candidum* was stored at −80°C and freeze-dried to produce a powder. A twenty-fold volume of boiling water was added to the powdered sample and the sample was extracted twice by stirring overnight. The aqueous extract was evaporated and concentrated using a rotary evaporator (N-1100; Eyala, Tokyo, Japan).

### Animals

Seven-week-old female ICR mice (n=100) were purchased from the Experimental Animal Center of Chongqing Medical University (Chongqing, China). The mice were maintained in a temperature- and humidity-controlled (temperature 25±2°C, relative humidity 50±5%) facility with a 12-h light/dark cycle and free access to a standard rat chow diet and water. All experimental procedures carried out in the present study were approved by the Ethics Committee of Chongqing Medical University

### Component analysis by nuclear magnetic resonance (NMR)

Dried *D. candidum* was refluxed and extracted 3 times with a 10-fold quantity of ethyl acetate. An ethyl acetate extract was obtained after 1 h for every reflux extraction and concentrated by evaporation under reduced pressure. The combined ethyl acetate extracts were extracted with anhydrous ethanol 3 times. The ethanol extract was suspended in water, and extracted by petroleum ether, chloroform and butanol extraction, respectively. The ethyl acetate extract was treated by gradient elution in a silica gel (Shanghai Xinhuo Silica Gel Factory, Shanghai, China) column with a petroleum ether-ethyl acetate solvent system. Then, the chloroform extract was treated by gradient elution in a silica gel column with a petroleum chloroform-methanol system. The butanol extract was treated with water with ultrasonic irradiation, and the extracting solution was isolated after filtering. The extract was then eluted using an HP2MGL macroporous resin (Mitsubishi Chemical Corporation, Tokyo, Japan) column with water, 10% ethanol, 30% ethanol and 60% ethanol, respectively. After elution, the various solvents contained different compounds, and their compositions were determined by NMR (Varian Inova 400; Varian Inc., Palo Alto, CA, USA). NMR was conducted using the following settings: ^1^H frequency, 300 MHz; temperature, 25°C; pulse length, 8 μsec; spin speed, 20 Hz; and scan number, 64 times. The ^1^H NMR spectra were recorded using a standard high-resolution magic angle spinning probe with magic-angle gradient.

### Induction of constipation in mice

To investigate the preventive effects of *D. candidum* against activated carbon-induced constipation, the animals were divided into five groups with 20 mice in each. The experimental design was as follows: the normal and control groups were fed a normal diet for 9 days; the high and low concentration *D. candidum* groups received 400 and 200 mg/kg body weight of the aqueous extract orally in a volume of 2 ml; and the drug cure group mice were treated with a 100 mg/kg dose of bisacodyl dissolved in water for 9 days. The control and treatment groups received an oral administration of activated carbon (0.2 ml of 10% activated carbon, w/w; activated carbon dissolved in 10% arabic gum) at 18:00 from the sixth to ninth day to induce constipation (11). The body weight, stool weight and stool moisture content were determined at 09:00 every day.

### Measurement of the defecation status of the mice

This measurement was performed to determine whether the prokinetic action of *D. candidum* was capable of propagating a prokinetic signal along the length of the GI tract. The excreted fecal pellets of individual mice were collected daily at 09:00 for the duration of the experiment. The total number, weight and water content of the pellets were determined. The water content was calculated as the difference between the wet and dry weight of the pellet. After 16 h, the mice in the control and treatment groups received 10% activated carbon and the normal group was administered 10% arabic gum by intragastric gavage. The animals were then placed individually in small transparent cages and allowed access to food and tap water *ad libitum*. The length of time from carbon meal administration to the appearance of darkened feces was recorded. Feces were collected, counted, weighed and their water content was evaluated.

### GI transit and defecation time

Mice were fasted for 16 h from the ninth day at 18:00; however, they were not deprived of water. After 16 h, the mice in the control and treatment groups received an oral administration of 10% activated carbon while the mice in the normal group received an oral administration of 10% arabic gum. After 30 min, the mice were sacrificed by cervical dislocation under anesthesia with diethyl ether. A total of 10 mice in each group were dissected and the small intestine from the pylorus to the cecum was carefully removed. The GI transit of each mouse was calculated as the percentage of the distance traveled by the activated carbon meal relative to the total length of the small intestine. The following equation was used to calculate GI transit: GI transit (%) = distance traveled by the activated carbon/total length of the small intestine ×100. The remaining 10 mice of each group were used to measure the time until the first defecation of the black stool following the oral administration of 10% activated carbon.

### Levels of MTL, Gas, ET, SS, AChE, SP and VIP in the serum

The levels of MTL, Gas, ET, SS, AChE, SP and VIP in the serum were determined using radioimmunoassay kits (Beijing Puer Weiye Biotechnology Co., Ltd., Beijing, China). The serum was collected from the heart following surgery.

### Statistical analysis

Data are presented as mean ± standard deviation (SD). Differences between the mean values for individual groups were assessed by one-way analysis of variance (ANOVA) with Duncan’s multiple range test. P<0.05 was considered to indicate a statistically significant difference. SAS version 9.1 (SAS Institute Inc., Cary, NC, USA) was used to conduct the statistical analyses.

## Results

### Constituents of D. candidum leaf

Eleven compounds were isolated and identified in *D. candidum* leaf. Compound 1 was obtained as a clear crystal; the ^1^H-NMR spectrum of this compound exhibited peaks at δ 6.92 (2H, d), 6.62 (2H, d), 6.06 (2H, s), 6.03 (1H, s) and 2.65 (4H, m), consistent with this compound being dihydro-resveratrol. Compound 2 was obtained as a white powder; the ^1^H-NMR spectrum of this compound exhibited peaks at δ 6.98 (2H, d), 6.74 (2H, d), 6.62 (1H, s), 6.47 (1H, d), 4.83 (1H, d), 4.63 (1H, d), 3.1–3.8 (12H), 3.73 (3H, s), 3.69 (3H, s) and 2.74 (4H, m), indicating that it was dendromoniliside E. Compound 3 was obtained as a black red needle; the ^1^H-NMR spectrum of this compound exhibited peaks at δ 11.00 (1H, s), 8.15 (1H, d), 6.06 (2H, s), 8.07 (1H, d), 6.95 (1H, s), 6.83 (1H, s), 6.15 (1H, s), 3.96 (3H, s) and 3.93 (3H, s), indicating that it was denbinobin. Compound 4 was obtained as a colorless needle; the ^1^H-NMR spectrum of this compound exhibited peaks at δ 4.72 (2H, m), 3.85 (1H, d), 6.06 (2H, s), 2.53 (1H, d), 2.49 (1H, t), 2.39 (1H, dd), 2.21 (1H, dd), 1.64 (1H, m), 1.35 (3H, s), 1.03 (3H, d) and 0.95 (3H, d), which suggests that this material was aduncin. Compound 5 was obtained as a white needle; the ^1^H-NMR spectrum of this compound exhibited peaks at δ 8.25 (1H, s), 8.10 (1H, s), 5.90 (1H, d), 4.66 (1H, dd) and 3.5–4.2 (4H, m), and it was confirmed that material this was adenosine. Compound 6 was obtained as a white powder; the ^1^H-NMR spectrum of this compound exhibited peaks at δ 7.95 (1H, d), 5.85 (1H, d), 5.66 (1H, d) and 3.2–4.3 (5H, m), confirming that the material was uridine. Compound 7 was obtained as a clear crystal; the ^1^H-NMR spectrum of this compound exhibited peaks at δ 10.60 (1H, s), 7.92 (1H, s), 6.45 (2H, s), 5.66 (1H, d), 3.4–4.4 (5H, m), indicating that the material was guanosine. Compound 8 was obtained as a white powder; the ^1^H-NMR spectrum of this compound exhibited peaks at δ 7.65 (1H, d), 7.41 (2H, d), 6.85 (2H, d), 6.33 (1H, d), 4.17 (2H, t), 1.69 (2H, m), 1.25 (54H, m) and 0.85 (3H, t), indicating that this material was defuscin. Compound 9 was obtained as a white powder; the ^1^H-NMR spectrum of this compound exhibited peaks at δ 7.45 (2H, d), 6.82 (2H, d), 6.81 (1H, d), 5.83 (1H, d), 4.16 (2H, t), 1.67 (2H, m), 1.23 (54H, m) and 0.88 (3H, t), indicating that this material was *n*-triacontyl *cis-p*-coumarate. Compound 10 was obtained as a white powder; the ^1^H-NMR spectrum of this compound exhibited peaks at δ 2.35 (2H, t), 1.62 (2H, m), 1.25 (24H, m) and 0.88 (3H, t), consistent with the material being hexadecanoic acid. Compound 11 was obtained as a white powder; the ^1^H-NMR spectrum of this compound exhibited peaks at δ 3.85 (2H, t), 1.75 (2H, m), 1.45 (2H, m), 1.22 (54H, m) and 0.85 (3H, t), and was identified to be hentriacontane.

### Body weight during the experiment

**Figure 1:**
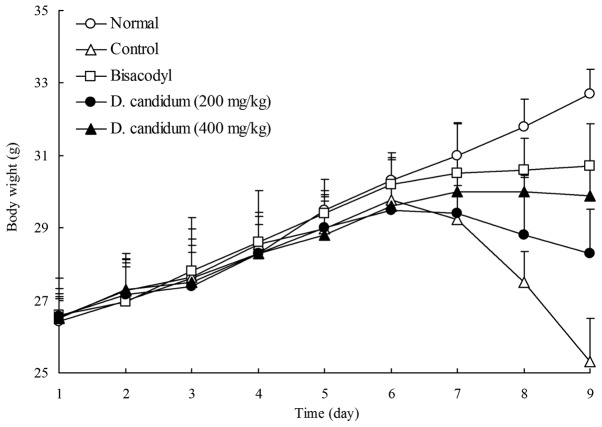
Body weight of mice during the experiment (n=10 ICR mice per group). The mice were treated with *Dendrobium candidum* Wall. ex Lindl (*D. candidum*) aqueous extract (200 or 400 mg/kg body weight) or bisacodyl at a dosage of 100 mg/kg body weight.

The body weights of the control mice with activated carbon-induced constipation were significantly decreased after six days. As shown in Fig. 1, following the initiation of activated carbon-induced constipation, the body weights of the mice in the *D. candidum-*treated groups were significantly lower compared with those of the normal mice and the bisacodyl-treated group, but higher than those of the control mice with activated carbon-induced constipation. The high dose of 400 mg/kg *D. candidum* alleviated the weight loss of the mice to a greater extent than the lower 200 mg/kg dose.

### Effect of D. candidum on defecation status of mice

From the first to the sixth day, defecation weight, particle counts of defecation and water content of defecation in each group were not significantly different, although the defecation weight and particle counts of defecation in the bisacodyl group were slightly greater than those in the other groups (Table I). When constipation was induced, from the seventh day to the ninth day, the defecation weight, particle counts of defecation and water content of defecation were decreased to 0.31 g, 16 pieces and 18%, respectively, in the control group. Particle counts were decreased to 0.78 g (32 pieces), 0.58 g (22 pieces) and 0.67 g (27 pieces), respectively, and the water content of defecation was decreased to 41, 26 and 35% in the bisacodyl and 200 and 400 mg/kg *D. candidum* dose groups, respectively. These results demonstrated that *D. candidum* was able to relieve constipation and had a good effect in the treatment of constipation.

### Time taken to the first defecation of a black stool

**Figure 2:**
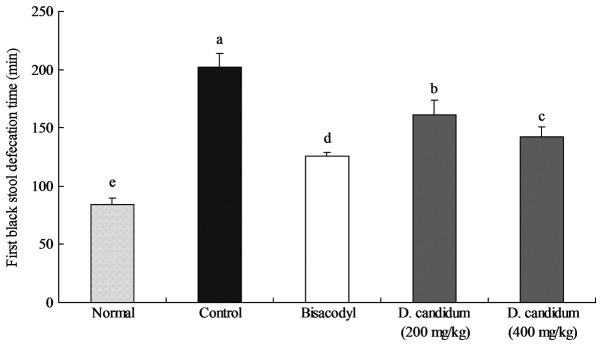
First black stool defecation time of mice following the induction of constipation by activated carbon at the last day in the experiment (n=10 ICR mice per group). The dose of bisacodyl was 100 mg/kg body weight. ^a–e^Mean values with different letters over the bars are significantly different (P<0.05) according to Duncan’s multiple range test.

The time taken for the first defecation of a black stool in each group of mice following the administration of activated carbon, which demonstrates the constipation-inhibiting effect of the different treatments, is shown in Fig. 2. The defecation time was the shortest (84±6 min) in the normal group and the longest (202±12 min) in the control group; the defecation time in the bisacodyl group was 126±3 min, higher only than that of the normal group. The times taken for the first defecation of a black stool for the mice treated with 200 and 400 mg/kg *D. candidum* were 161±9 and 142±6 min, respectively. According to the defecation time, *D. candidum* demonstrated a strong effect as an inhibitor of constipation.

### GI transit

**Figure 3:**
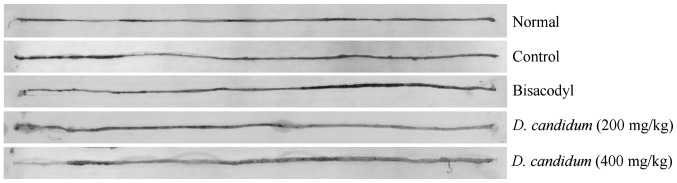
Effects of samples on gastrointestinal (GI) transit in activated carbon-induced constipation model mice. n=10 ICR mice in each group, bisacodyl: 100 mg/kg b.w. ^a–e^Mean values with different letters over the bars are significantly different (P<0.05) according to Duncan’s multiple range t.

The constipation-inhibiting effects of the treatments were determined by GI transit in mice following the administration of activated carbon (0.2 ml/mouse, 10% activated carbon). In the bisacodyl-treated group, the mean GI transit was 90.2±4.8%, which was higher than that of the control group (48.8±6.1%; Fig. 3). The GI transits of the mice in the 200 and 400 mg/kg *D. candidum* treatment groups were 57.7±4.6 and 74.6±2.8%, respectively. *D. candidum* increased the GI transit compared with that of the control, reduced constipation and had an increased functional effect on GI transit.

### MTL, Gas, ET, SS, AChE, SP and VIP levels in the serum

Normal mice showed the highest MTL, Gas, ET, AChE, SP and VIP levels; however, these levels in the control mice were significantly decreased (P<0.05, Table II). The levels of these analytes in the bisacodyl-treated mice were most similar to those of the normal mice. In the *D. candidum*-treated mice, these analyte levels were also comparable with those in the normal mice and higher than those of the control mice. Additionally, the mice treated with 400 mg/kg *D. candidum* exhibited higher levels than did the mice treated with 200 mg/kg *D. candidum*. The higher dose helped to promote these analyte levels to similar values as were observed in the normal and bisacodyl-treated mice. The SS levels in mice showed the opposite tendency.

## Discussion

The definition of constipation includes infrequent bowel movements and difficulty during defecation (12,13). Constipation most commonly occurs when the stool that forms after food is digested moves too slowly as it passes through the digestive tract. Dehydration, changes in diet and activity, and certain drugs are frequently responsible for the slow transit of stools. When stools move slowly, too much water is absorbed from the stool and it becomes hard and dry (14). Defecation status, stool defecation time and GI transit are important standards when investigating constipation.

The serum levels of MTL, Gas, ET, AChE, SP and VIP in patients with constipation are lower than those in healthy individuals while the SS levels are higher (15–17). The main function of MTL is to increase the migrating myoelectric complex component of GI motility and stimulate the production of pepsin. It is one of the intestinal hormones responsible for the proper filling and emptying of the GI system in response to food intake, as well as hunger stimuli and responses (18). Gas is a polypeptide hormone secreted by certain cells of the pyloric glands, which strongly stimulates the secretion of gastric acid and pepsin, and weakly stimulates the secretion of pancreatic enzymes and gallbladder contraction (15). Gas produces effects throughout the GI tract, including promoting GI secretion, increasing GI movement and promoting pyloric sphincter relaxation. ET plays an important role in the stability of vascular tension and maintains the basic cardiovascular system. Constipation, in addition to causing intestinal obstruction, also induces or aggravates cardiocerebrovascular diseases in the elderly (19). An SS analog, octreotide, has been reported to stimulate intestinal motor complexes and this agent has been used to treat sclerodermatous pseudo-obstruction (20). Stools are formed from the non-digestible components of food after water is either absorbed or secreted in the large intestine. Mucous is also produced in the large intestine to provide viscosity. Thin segments of muscle line the intestinal tract and contract and relax in concert to propel the stool forward. Muscle contraction and mucous secretion are regulated by acetylcholine (21). Patients with slow-transit constipation have abnormalities of the neurotransmitters in the muscular layer of their intestinal walls. These abnormalities include a deficiency of a peptide known as SP, which is thought to contribute to peristalsis (22). Disturbances in the normal neural content of VIP in the bowel wall in idiopathic constipation and diverticular disease may initiate or contribute to the functional changes observed in these disorders (23).

*D. candidum* has been used as a traditional Chinese medicine. It has been reported to have various therapeutic effects on numerous pathologic conditions such as inflammation, immunity, hyperglycemic and cancer (24). However, to the best of our knowledge, there are no scientific studies concerning its anti-constipation effects. In the present study, 11 compounds were isolated from the leaf of *D. candidum*. These compounds might possess anti-constipation activities and, with the high content of functional compounds, may be the reason why *D. candidum* demonstrated good functional effects in the prevention of constipation. In addition, the synergistic activities of the various bioactive components may increase the anti-constipation effect of *D. candidum*. Resveratrol has been recommended for the prevention of constipation (25), and adenosine A1 receptor antagonists have been suggested to be of therapeutic potential in constipation (26). Uridine, guanosine, hexadecanoic acid and hentriacontane have also demonstrated an association with decreased constipation (27–29). Aduncin, a component only found in *Dendrobium*, may have constipation-preventing effects; however, its functional effects require investigation (30). Defuscin, *n*-triacontyl *cis-p*-coumarate, hexadecanoic acid and hentriacontane have also demonstrated showed numerous functional activities in human health (31,32).

In summary, the present study found that *D. candidum* has potent *in vivo* anti-constipation activities, provided by various functional components. The scientific data concerning serum content, defecation status, GI transit and defecation time demonstrated the functional effects and provide a scientific basis for the development of *D. candidum*.

## Figures and Tables

**Table I:** Defecation status of the various groups of mice during the experiment.

				*D. candidum* (mg/kg)	
				
Treatment	Normal	Control	Bisacodyl	200	400
Days 1–6^a^					
Defecation weight (g)	0.92±0.07^d^	0.93±0.08^d^	1.04±0.08^c^	0.94±0.03^d^	0.96±0.06^d^
Particle counts of defecation	34±5^d^	35±3^d^	44±3^c^	35±4^d^	36±4^d^
Water content of defecation (%)	45±5^d^	46±3^d^	50±6^c^	45±5^d^	47±4c,d
Days 7–9^b^					
Defecation weight (g)	0.93±0.06^c^	0.31±0.03^g^	0.78±0.11^d^	0.58±0.03^f^	0.67±0.05^e^
Particle counts of defecation	35±4^c^	16±5^g^	32±4^d^	22±4^f^	27±5^e^
Water content of defecation (%)	46±3^c^	18±5^g^	41±5^d^	26±5^f^	35±6^e^

a Treatment alone was administered;

b treatment and activated carbon were administered. Values presented are the mean ± standard deviation (n=10 ICR mice per group). The dosage of bisacodyl was 100 mg/kg body weight.

c–g Mean values with different letters in the same column are significantly different (P<0.05) according to Duncan’s multiple range test.

**Table II:** Effect of *D. candidum* on serum MTL (motilin), Gas (gastrin), ET (endothelin), SS (somatostatin), AchE (acetylcholine enzyme), SP (substance P) and VIP (vasoactive intestinal peptide) levels in activated carbon-induced constipation model mice.

				*D. candidum* (mg/kg)	
				
Analyte (pg/ml)	Normal	Control	Bisacodyl	200	400
MTL	166.3±10.8^a^	87.2.3±9.2^e^	150.2±8.2^b^	111.7±8.7^d^	142.6±7.1^c^
Gas	83.1±3.8^a^	40.4±4.1^e^	70.2±3.1^b^	53.1±2.1^d^	66.1±3.1^c^
ET	14.6±0.6^a^	6.4±0.5^e^	12.6±0.5^b^	7.4±0.4^d^	11.7±0.2^c^
SS	34.1±.1.2^e^	69.2±4.3^a^	38.9±2.1^d^	50.8±2.2^b^	41.1±0.3^c^
AchE	33.8±1.4^a^	11.6±0.3^e^	28.2±1.1^b^	17.7±0.8^d^	24.2±0.3^c^
SP	69.2±3.1^a^	31.7±1.1^e^	62.1±1.5^b^	47.1±1.5^d^	57.1±1.3^c^
VIP	56.2±1.3^a^	32.6±1.3^e^	51.2±1.8^b^	38.2±1.6^d^	45.5±1.1^c^

Values presented are the mean ± standard deviation (n=10 mice per group). MTL, motilin; Gas, gastrin; ET, endothelin; SS, somatostatin; AChE, acetylcholinesterase; SP, substance P; VIP, vasoactive intestinal peptide. The dose of bisacodyl was 100 mg/kg body weight.

a–e Mean values with different letters in the same column are significantly different (P<0.05) according to Duncan’s multiple range test.
